# Epidemiological analysis of single center of acute poisoning cases based on poisoning treatment platform

**DOI:** 10.1097/MD.0000000000026444

**Published:** 2021-07-09

**Authors:** Shen Jun-hua, Zhang Jin-song, Qiao Li, Wang Lei, Zhu Bao-feng, Li Jia, Liu Ying, Cheng Jian-Rong

**Affiliations:** a The Second Affiliated Hospital of Nantong University; b The first Affiliated Hospital with Nanjing Medical University, China.

**Keywords:** acute poisoning, epidemiology, poisoning treatment platform, prospective

## Abstract

To studied epidemiological characteristics of 493 cases of acute poisoning in Nantong city, Jiangsu province.

Based on the analysis platform of poisoning treatment, adopted single center and prospective investigation method, analyzed data of acute poisoning patients from May 2015 to December 2018 in the second affiliated hospital of Nantong University.

Among 493 patients with acute poisoning, men 227 (46.04%), women 266 (53.96%). Age ranged from 12 to 89 years old, average age 41.6 years. In the occupational distribution, farmers were 30.02%; 351 cases (71.20%) visited the hospital within 6 hours after exposure. Oral exposure poisoning 415 cases (84.18%). Pesticide poisoning accounted for 45.45% of deaths.

Using the poisoning treatment platform to analyze the clinical characteristic had accurately and reliably in Nantong. The fatality rate of pesticide poisoning in cases of acute poisoning is high. Management of highly toxic pesticides should be continued and effective health education on pesticide use should be carried out.

## 1 Introduction

Acute poisoning accounted for 5.6% of the patients in Emergency department (ED), and the trend of increasing gradually. According to National Health Commission of the People's Republic of China, poisoning and injury have become the top 5 causes of death in China, accounting for 10.7% of the total mortality rate.^[1,2]^ Acute poisoning was also one of the most common critical cases in emergency, with the development of social economy, the progress of industry, the change of human life style. The spectrum, population, treatment of acute poisoning have changed obviously.^[3,4]^ Based on the poisoning treatment platform, this study adopted the prospective research method, then investigate and analyzed the epidemiological characteristics of poisoning in Nantong city, Jiangsu province.

## 2 Methods

### 2.1 General data

Clinical data onto acute poisoning patients in the ED of the second affiliated hospital of Nantong university from May 2015 to December 2018 were selected, and excluded poisoning cases of children under 12 years old.

### 2.2 Ethics and informed consent

This study was approved by the Ethics Committee of The Second Affiliated Hospital of Nantong University (IRB number: 2015ky013).

### 2.3 Statistical analysis

Adopt prospective investigation method, application “hospital acute poisoning case information management system” (Sue ICP for 16042896), based on a Chinese slow disease network platform, design the questionnaire of acute poisoning, including sex, age, occupation, vital signs, types of poison, poison contact time, cause of poisoning, poisoning and dosage, and accept the gastric lavage, antidote, glucocorticoid treatment, after treatment outcome, etc. To describe the continuous and qualitative variables, mean standard deviation and frequency (%) were reported.

## 3 Results

### 3.1 General situation

Among the 493 patients with acute poisoning, men 227 (46.04%) and women 266 (53.96%). The age from 12 to 89 years old, average age 41.6 years. Thirty to 39 years group most poisoning cases (18.05%). (Table 1).

**Table 1:** Age distribution of acute poisoning cases.

Age, yr	n	(%)
12–19	25	5.07
20–29	87	17.65
30–39	89	18.05
40–49	81	16.43
50–59	87	17.65
60–69	54	10.95
70–79	41	8.32
80–89	29	5.88
Total	493	100

### 3.2 Occupational distribution

Among the 493 patients with acute poisoning, farmers were 30.02%, followed by individual industrial and commercial was 15.01% (Table 2).

**Table 2:** Occupational distribution of acute poisoning.

Occupational	n	(%)
Farmers	148	30.02
Individual industrial and commercial	74	15.01
Business services	62	12.58
Chemical enterprises workers	40	8.11
Building materials processing workers	35	7.10
Retired personnel	32	6.49
Administrative personnel	24	4.87
Students	18	3.65
Driver	14	2.84
Construction worker	10	2.03
Medical	6	1.22
Fishermen	6	1.22
Teacher	4	0.81
Homemaker	4	0.81
Agricultural technology personnel	3	0.61
Other	12	2.43
Total	493	100

### 3.3 Visit time after exposure to poisoning

Three hundred fifty one cases (71.20%) visited the hospital within 6 hours after exposure, 89 cases (18.05%) visited the hospital within 6 to 24 hours after exposure, 53 cases (10.75%) visited the hospital longer than 24 hours after exposure.

### 3.4 Poisoning pathways

Among the 493 patients with acute poisoning, oral exposure poisoning 415 cases (84.18%), respiratory exposure 68 cases (13.79%) (Table 3).

**Table 3:** Distribution of poisoning pathways.

Poisoning pathways	n	(%)
Respiratory exposure	68	13.79
Skin absorb	8	1.62
Intravenous	2	0.41
Oral exposure	415	84.18
Total	493	100

### 3.5 Poison species

Among the 493 patients with acute poisoning, pesticide poisoning 178 cases (36.11%), drug poisoning 158 cases (32.05%) (Table 4).

**Table 4:** Species distribution of acute poisons.

Poison species	n	(%)
Pesticide	178	36.11
Drug	158	32.05
Food-borne	35	7.10
Chemical poisons	60	12.02
Other classes	42	8.52
Unknown reasons	20	4.06
Total	493	100

### 3.6 Food-borne poisons classification

Among the 35 patients with food-borne poisoning, alcohol poisoning 21 cases (60.0%), animal and plant poisoning 8 cases (22.86%) (Table 5).

**Table 5:** Classification of food-borne poisons.

Classification	n	(%)
Alcohol poisoning	21	60.00
Nitrite poisoning	2	5.71
Animal and plant poisoning	8	22.86
other	4	11.43
Total	35	100

### 3.7 Drug poisons classification

Among the 158 cases of drug poisoning, sedative-hypnotic drugs 93 cases (58.86%), antipsychotic drugs 16 cases (10.13%) (Table 6).

**Table 6:** Classification of drug poisons.

Drug	n	(%)
Sedative hypnosis	93	58.86
Antipsychotic	16	10.13
Cardiovascular drugs	10	6.33
Nonsteroidal anti-inflammatory drugs	6	3.80
Digestive system drug	2	1.27
Traditional Chinese medicine	1	0.63
Other	25	15.82
Did not fill	5	3.16
Total	158	100

### 3.8 Pesticide poisons classification

Among the 178 cases of pesticide poisoning, organophosphorus 88 cases (49.44%), pyrethroids 26 cases (14.61%) (Table 7).

**Table 7:** Classification of pesticide poisons.

Pesticide	n	(%)
Organophosphorus	88	49.44
Pyrethroids	26	14.61
Herbicide	25	14.04
Rodenticide	19	10.67
Chlordimeform	3	1.69
Abamectin	3	1.69
Carbamate	1	0.56
Organochlorine	1	0.56
Other	7	3.93
Did not fill	5	2.81
Total	178	100

### 3.9 Chemical poisons classification

Among the 60 cases of chemical toxicosis, nitrite poisoning 6 cases (10%), organic solvents poisoning 4 cases (6.67%) (Table 8).

**Table 8:** Classification of chemical poisons.

Chemical poisons	n	(%)
Nitrite poisoning	6	10.00
Organic solvent poisoning	4	6.67
Acid alkali	2	3.33
Cyanide poisoning	1	1.67
Other chemical poisons	47	78.33
Total	60	100

### 3.10 Gastric lavage analysis of oral poisoning

Among the 415 cases of oral exposure poisoning, gastric lavage 155 cases (37.35%), time to start gastric lavage <30 minutes 99 cases (63.87%), gastric lavage juice volume 10 to 20 L 122 cases (78.71%) (Table 9).

**Table 9:** Gastric lavage analysis of oral poisoning.

Gastric lavage therapy	n	(%)
Time to start gastric lavage		
<30 min	99	63.87
30–60 min	49	31.61
60–120 min	7	4.52
Gastric lavage juice volume		
<10 L	5	3.23
10–20 L	122	78.71
20–50 L	27	17.42
>50 L	1	0.65
Total	415	100

### 3.11 Treatment of acute poisoning

Among the key rescue treatments for patients with acute poisoning, 105 cases (21.29%) of specific antidote, including atropine, naloxone, melanin, flumazenil, vitamin K1, cholinesterase reactivator, etc. Fifty seven patients (11.56%), including methylprednisolone, dexamethasone, and hydrocortisone. Thirty eight cases (7.7%) of blood purification treatment, including hemoperfusion, hemodialysis, etc. Thirty seven cases (7.51%) of hyperbaric oxygen treatment. All patients with poisoning received symptomatic supportive treatment (Table 10).

**Table 10:** Main treatment measures for patients with acute poisoning.

Treatment	n	(%)
Special antidote	105	21.29
Glucocorticoid	57	11.56
Blood purification	78	15.82
High pressure	37	7.51
Total	277	56.18

### 3.12 Outcomes and follow-up

We followed up to the end of December 2019 after all patients were actively treated. Three hundred ninety three patients (79.72%) were cured of acute poisoning, including 279 patients (56.59%) hospitalized, 114 patients (23.12%) in ED, and 89 patients (18.05) with residual organ dysfunction, including nervous system and respiratory system. There were 11 deaths (2.23%), including 5 cases of pesticide poisoning (3 cases of organophosphorus, 2 cases of Paraquat ), 2 cases of sedative sleep aids, 2 cases of alcoholism, 1 case of carbon monoxide poisoning, and 1 case of unknown poison (Table 11).

**Table 11:** Outcomes of patients with acute poisoning.

Outcome	n	(%)
Heal	393	79.72
Organ dysfunction	89	18.05
Death	11	2.23
Total	493	100

## 4 Discuss

This research hospital is a designated acute poisoning treatment center in Nantong city. The included subjects are from Nantong city and surrounding counties, which can reflect the epidemiological characteristics of poisoning in Nantong city. Based on “hospital acute poisoning case information management system,” the data are true and reliable.

Among the 493 cases of acute poisoning, women poisoning were 266 (53.96%), then higher than that men poisoning. Similar to reports by Qiao et al,^[5]^ which may be related to women's occupation, education level, economic income, psychological factors.^[6]^ From the analysis of poisoning age, most poisoning cases are concentrated in adults, 30 to 39 years age group has the most poisoning cases, 20 to 29 years old and 40 to 49 years old is similar number. More than 80 years old account for 5.88%, the higher than those reported by Wang et al^[9]^ and Li et al,^[4]^ etc, which are considered to be related to the aging process of Chinese society, Nantong is a famous hometown of longevity.

Among the causes of poisoning exposure, suicide is the most common, oral poisoning is the most common poisoning pathways, the most common poison is pesticides. This study found that organophosphorus pesticides accounted for 49.44% of all pesticide poisons, followed by pyrethroids, herbicides, and rodenticides.

At present, there is still a lack of evidence-based medical evidence about gastric lavage treatment, and there is some controversy.^[7]^ There are some unreasonable phenomena in clinical gastric lavage operation, such as excessive gastric lavage, repeated gastric lavage, and inadequate respiratory protection during gastric lavage.^[2]^ However, at the present stage, gastric lavage is still the most common method for emergency oral poisoning to remove toxic substances.^[8]^ In this study, 37.35% of patients with oral toxic exposure received gastric lavage. The application of blood purification in patients with acute poisoning is significant, effectively improving the clinical treatment of patients,^[10]^ promoting faster recovery of patients, significantly reducing mortality, and has higher clinical application value.

In this study, 79.72% of patients were cured and 2.23% of patients died. Five of the deaths were pesticide poisoning, accounting for 45.45% of the deaths. At present, respiratory failure and myocardial damage caused by Organophosphorus are the main causes of death. At present, the toxicological mechanism of Paraquat is unknown, the lethal dose is low, the mortality rate is extremely high, and there is no effective antidote. In recent years, the application of new pesticides, especially compound pesticides, has lacked detailed instructions and specific ingredients could not be clarified, which has brought challenges to diagnosis and treatment. Pesticide poisoning is still a major problem in Jiangsu province,^[11,12]^ and the management of highly toxic pesticides should be continued carry out effective health education on pesticide use. Strengthen their awareness of pesticide regulation.

As a single center study, this study has some limitations. There are some differences in the clinical treatment of acute poisoning in economically developed and economically underdeveloped regions and in different cultural regions in China.^[2,10]^ In the future, we will carry out multi-center research on the basis of this study, then it will help promote the poisoning treatment in Nantong.

## Author contributions

**Conceptualization:** Zhang Jin-song.

**Data curation:** Zhu Bao-feng.

**Formal analysis:** Qiao Li.

**Methodology:** Wang Lei, Li Jia.

**Project administration:** Liu Ying.

**Writing – original draft:** Shen Jun-hua.

**Writing – review & editing:** Cheng Jian-Rong.

## References

[1] Chu P Zhang YM Gao YL. Current situation and development of clinical treatment of acute poisoning in China. J Clin Emerg 2013;14:455–8.

[2] Ma JF Liao CJ. Analysis of the factors influencing the prognosis of acute poisoning patients in emergency medicine and the countermeasures. Chin Commun Phys 2019;35:42–4.

[3] Bundotich JK Gichuhi MM. Acute poisoning in the Rift Valley Provincial General Hospital, Nakuru, Kenya: January to June 2012. S Afr Fam Pract 2015;57:01–5.

[4] Li ZH Wang GX Zhen GD Zhang Y Liu J Liu S. Application of hemoperfusion in severe acute organophosphorus pesticide poisoning. Turk J Med Sci 2017;47:1277–81. 29156874 10.3906/sag-1611-40

[5] Qiao L Zhang JS Chen JR. A multicenter prospective clinical epidemiological study of 1965 cases of acute poisoning. Chin J Emerg Med 2016;25:1376–80.

[6] Zhu BF Chen JR. Epidemiological investigation and analysis of 87 cases of acute pesticide poisoning. Hainan Med J 2012;23:134–5.

[7] Qiao L Zhang JS. Current situation and thinking of clinical treatment of acute poisoning in China. Chin J Emerg Med 2015;24:1193–6.

[8] Sun QS Sun H Zhao HM. A prospective clinical epidemiological study of 424 cases of acute poisoning. J Clin Emerg 2018;19:346–50.

[9] Wang BS Chen L Li XT. Acute Pesticide Poisoning in Jiangsu Province, China, from 2006 to 2015. Biomed Environ Sci 2017;30:695–700. 29081347 10.3967/bes2017.094

[10] Zhang S Jian XD Yang KC. Epidemiological and psychological analysis of 220 patients with acute self poisoning. Occup Health Emerg Rescue 2019;37:353–5.

[11] Yang X Xin S Zhang Y. Early hemoperfusion for emergency treatment of carbamazepine poisoning. Am J Emerg Med 2018;36:926–30. 29066188 10.1016/j.ajem.2017.10.048

[12] Wang N Jiang Q Han L. Publisher correction: epidemiological characteristics of pesticide poisoning in Jiangsu Province, China, from 2007 to 2016. Sci Rep 2019;9:1879. 31197201 10.1038/s41598-019-44986-7 PMC6565669

